# Emergency surgery, acute care surgery and the boulevard of broken dreams

**DOI:** 10.1186/1749-7922-4-4

**Published:** 2009-01-29

**Authors:** Fausto Catena, Ernest E Moore

**Affiliations:** 1 General, Emergency and Transplant Surgery DPT, St Orsola-Malpighi University Hospital Bologna, Bolgna, Italy; 2 Department of Surgery, Denver Health Medical Center, University of Colorado Health Sciences Center, Denver, USA

The scenario sounds familiar. Last night I returned home very late after a full day of emergency surgeries. While I was having my usual cold dinner in front of the television with my entire family asleep, I received disturbing news; a famous Italian actor had undergone emergency surgery in Honduras. the actor had been participating in a "reality show" and experienced severe back pain. For this reason, he was treated for one week with a NSAID. Suddenly, he developed acute abdominal pain and was taken to the nearest hospital. The diagnosis was acute appendicitis and he underwent immediate laparotomy via a Mc Burney incision. Unfortunately, the surgeons were mistaken. The problem was a large perforated duodenal ulcer and the actor then underwent a midline incisioThe patient was subsequently transferred to a Miami acute care hospital.

The story is always the same; we can call it emergency surgery, acute care surgery, or "Samantha," but the key point is that we do not have a widespread set of minimum standards for emergency surgery.

Such standards are just as important as those of ATLS.

We need to develop guidelines regarding organizational models to address diseases requiring urgent surgical intervention.

This is an integral component in the mission of the World Journal of Emergency Surgery and of the World Society of Emergency Surgery.

We must be uniformly prepared all around the world, similar to the uniform emergency protocols for airplanes and airports.

If we fail to meet this objective, we will continue to witness preventable complications and deaths affecting both the famous and the non-famous alike.

This is a dream, but it needn't be a broken one.

In 2010 we will have the 1^st ^World Congress of WSES. If we can successfully develop solid guidelines for surgeons from all around the world we will have accomplished a small yet important "humanitarian mission."

These guidelines would be freely available on the WJES web site and surgeons all around the world could reference them.

You can call it emergency surgery or acute care surgery, but not the "Boulevard of Broken Dreams".

